# Non-linear interaction modulates global extreme sea levels, coastal flood exposure, and impacts

**DOI:** 10.1038/s41467-020-15752-5

**Published:** 2020-04-21

**Authors:** Arne Arns, Thomas Wahl, Claudia Wolff, Athanasios T. Vafeidis, Ivan D. Haigh, Philip Woodworth, Sebastian Niehüser, Jürgen Jensen

**Affiliations:** 1 Faculty of Agricultural and Environmental Sciences, University of Rostock, Justus-von-Liebig-Weg 6, 18059 Rostock, Germany; 2 Department of Civil, Environmental and Construction Engineering, National Center for Integrated Coastal Research, University of Central Florida, 12800 Pegasus Drive, Suite 211, Orlando, FL 32816 USA; 3 Coastal Risks and Sea-Level Rise Research Group, Department of Geography, Christian-Albrechts-University Kiel, 24118 Kiel, Germany; 4 School of Ocean and Earth Science, National Oceanography Centre Southampton, University of Southampton, Waterfront Campus, European Way, Southampton, SO14 3ZH United Kingdom; 5 National Oceanography Centre, Joseph Proudman Building, 6 Brownlow Street, Liverpool, L3 5DA United Kingdom; 6 Research Institute for Water and Environment, University of Siegen, Paul-Bonatz-Str. 9-11, 57076 Siegen, Germany

**Keywords:** Environmental impact, Physical oceanography

## Abstract

We introduce a novel approach to statistically assess the non-linear interaction of tide and non-tidal residual in order to quantify its contribution to extreme sea levels and hence its role in modulating coastal protection levels, globally. We demonstrate that extreme sea levels are up to 30% (or 70 cm) higher if non-linear interactions are not accounted for (e.g., by independently adding astronomical and non-astronomical components, as is often done in impact case studies). These overestimates are similar to recent sea-level rise projections to 2100 at some locations. Furthermore, we further find evidence for changes in this non-linear interaction over time, which has the potential for counteracting the increasing flood risk associated with sea-level rise and tidal and/or meteorological changes alone. Finally, we show how accounting for non-linearity in coastal impact assessment modulates coastal exposure, reducing recent estimates of global coastal flood costs by ~16%, and population affected by ~8%.

## Abstract (web summary)

The non-linear interaction between tide and non-tidal residual impacts current and future extreme water levels. Here, based on 620 gauge records, the authors find a large non-linear interaction in the US East Coast, North Sea and parts of southern Japan, that results in a reduction of extreme sea levels.

## Introduction

Large parts of the world’s coastlines are periodically exposed to extreme sea levels caused by storm surges and high astronomical tides, which have the potential to cause widespread flooding and costly damages^1–3^. Knowledge of potential sea level extremes is thus essential for planning coastal protection strategies. Estimating the likelihood of extreme sea levels relies upon observations from tide gauges (see e.g., ref. ^4^). These instruments measure the local water level response to astronomical (i.e., tide) and meteorological forcing (i.e., surge, but also other contributions such as the regional wave set-up, see e.g., ref. ^4^) superimposed on the baseline mean sea level (e.g., ref. ^3^). For more than 60 years, nonlinear interactions between the tide and non-tidal components of sea level have been reported for many places around the world (e.g., refs. ^5–9^), resulting in the highest observed surges (defined by the residuals in a tidal analysis) occurring around mid-tide or low-tide rather than at the time of tidal high water^5–7^.

Previous work suggests that tide-surge interaction (TSI) cannot be neglected when reliable predictions of total water level extremes are needed (e.g., refs. ^5–9^), but a distinct global quantification is still missing. This shortcoming can be explained as follows. First, most research on TSI is local in character and findings can therefore not be transferred to other locations. Specifically, early studies on TSI focused on estuaries (refs. ^5,6,10–12^) or bays (refs. ^13,14^), followed by studies with emphasis on broad continental shelves including the southern North Sea (refs. ^15,16^), the UK coastline (ref. ^7^), the English Channel (refs. ^9,17^), and the Taiwan Strait (ref. ^8^), all identifying local characteristics and drivers. Second, other studies have largely focused on numerical model experiments enabling the separation of the contributions of tide, surge, and TSI (e.g., refs. ^15,18,19^) in order to obtain reliable information on their individual significance. Numerical models used in this way need to reproduce accurately tide and surge^9^, which are both strongly affected by bathymetric and geometric features^20^. These, however, are usually not fully resolved in numerical models due to limited details in the available datasets or the resolution of the computational grid^21^. Third, the existing numerical model experiments are limited to studies over a few storm events or days, and hence do not reflect the general underlying sea level system behaviour. Finally, explanations of physical drivers of TSI are mostly based on the equations of motion, including: advective terms, nonlinear bottom friction, and shallow water effects. However, none of the previous studies were able to provide a generalised explanation that was transferable to other sites around the world, as all of them focused on particular cases (e.g., event characteristics, direction and strength of the storm) and settings (e.g., topography, tidal range, tidal currents), marked by a varying importance of each of the three drivers (e.g., refs. ^7,10,15,18^).

TSI estimates are further hampered by methodological limitations that introduce artefacts that interfere with the natural dynamics. For example, ref. ^22^ discuss timing and analysis errors stemming from common harmonic analysis approaches that, in turn, also bias pure surge calculations. A more accurate term is thus the non-tidal residual (NTR), describing the signal that remains once the astronomical tide has been removed. A statistical dependence between tide and NTR is observed when decomposing total water levels into tide and NTR but conditional to the total water level percentile under investigation (Supplementary Fig. 1). Here, we continue using the established term tide-surge interaction but note that tide-NTR interaction is what we actually analyse.

Because of the issues discussed above, several studies have used skew surge (i.e., the difference between the maximum observed sea level around the astronomical high tide and the maximum predicted tidal level regardless of their timing) rather than the NTR (see e.g., ref. ^22^). For instance, ref. ^23^ showed that the 1% largest skew surges are independent of the tide for selected stations along the North Atlantic. However, we find that for the skew surges (or NTR) that have, combined with tide, led to the highest 1% of total water levels, dependence is significant between skew surge (or NTR) and tide (see Supplementary Fig. 2 and Supplementary Fig. 9).

Here, we build directly on existing studies and advance the estimation of TSI influences on extreme water levels by introducing a novel statistical method and applying this globally. In particular, we examine the dependence τ (i.e., Kendall’s rank correlation measuring the ordinal association, where 0 is no- and 1/−1 a perfect relationship/disagreement) between tide and NTR as observed at the peak high waters of total water levels. We use data from 621 individual tide gauge stations from a quasi-global tide gauge data set with high temporal resolution (i.e., at least hourly), called GESLA-2 (Global Extreme Sea Level Analysis)^24^ (https://www.gesla.org). Next, we use copula theory to identify an appropriate model to capture the dependence structure between tide and NTR and combine joint occurrences of the two variables to calculate total extreme sea levels (see Methods section). We repeat our analysis but assume tide and NTR are completely independent (i.e., τ_0_ = 0), i.e., NTR maxima can occur at any phase of the tide. A set of artificial total extreme sea levels is constructed from a randomised summation of observed tide and NTR (i.e., excluding the effect of TSI) and differences between both sets of extreme sea level are assumed to be caused by TSI. Consequently, we present a first of its kind analysis of TSI from multi-decadal tide gauge records and apply it globally. As secondary objectives, we investigate how TSI has changed over the last 60 years and discuss potential implications for future extreme sea levels. Finally, we compare TSI to recent sea-level rise (SLR) projections and estimate its importance for exposed coasts in terms of affected populations and flood costs.

## Results

### Spatial characteristics of non-linear effects

**Fig. 1:**
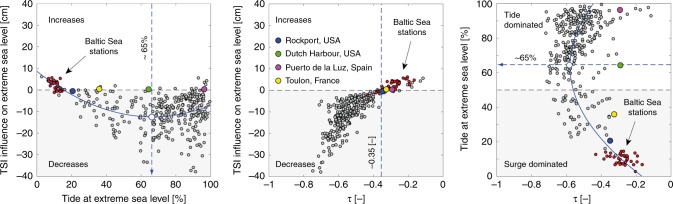
Max. non-linear effects on extreme sea levels. The figure indicates how non-linear changes extreme sea levels (defined here as the 99th percentile threshold exceedances) for (**a**) the entire World, (**b**) Europe, (**c**) Japan, and (**d**) the USA. Water level decreases from non-linear effects are shown in red (i.e., water levels are lower than from a linear superposition of tide and non-tidal residual alone) and increases in blue. Tidal range has been derived from OTIS tidal constituents.

The TSI influences on the 99th percentile of extreme sea levels (assumed here as quasi-extreme) are highlighted globally in Fig. 1a. The largest effects of TSI are found for the US East Coast and the Gulf of Mexico, the UK North Sea coastline, and parts of the southern Japanese coast. Along the US East Coast (see Fig. 1d), the average TSI effects on the 99th percentile extreme sea level amount to −28 cm (showing largest effects along the Middle Atlantic Bight with an average of −38 cm), a maximum of −61 cm at Sandy Hook (NY), and a minimum of −14 cm in Bar Harbour (ME). In the Gulf of Mexico, TSI effects are slightly less pronounced with an average of −27 cm, reaching values of −45 cm at Pensacola (FL) and −36 cm at St. Petersburg (FL). On the coasts of the Mid-Atlantic Bight and the Gulf of Mexico, average tidal contributions to extreme sea levels are 63 and 45% and are thus close to where largest TSI effects are expected from Supplementary Fig. 4d (see A simple proxy for non-linearity section). For comparison, the US West Coast is affected to a proportionately larger extent by the tides, accounting for 85% of the observed extreme sea levels but notably smaller TSI effects averaging to −10 cm.

Along the UK North Sea coastline (see Fig. 1b), the largest TSI effects are found to be −37 cm at North Shields increasing to −66 cm in Cromer and −55 cm in Dover, averaging at −48 cm for the mid-eastern to south-eastern UK coastline. Despite strong TSI effects, tidal contributions are large (average of 82%), again highlighting strong variations when using tides as a proxy for TSI. However, further southwest, i.e., in the English Channel and on the Brittany coast, the average tidal contribution and TSI effects are 88% and −42 cm at Dunkerque, 91% and −28 cm at Saint-Malo, and 96% and −18 cm at Le Conquet, showing a tendency towards reduced TSI effects associated with larger tidal contributions.

Along the southern Japanese coast (see Fig. 1c), a marked TSI effect with an average of −22 cm (71% tidal) is found. This area is characterised by a rugged coastline and stations with the largest TSI effects are located in marginal seas or bays. Specifically, at Nagoya TSI amounts to −50 cm (75% tidal); the station is located at the end of Ise Bay where water levels are potentially affected by geometric and bay effects. Similar influences are apparent at the stations Uno with a TSI of −42 cm (76% tidal) and Matsuyama with −39 cm (89% tidal), all of which are excluded from further analyses. After excluding them the average TSI drops slightly to −20 cm (70% tidal). Overall, our statistically derived TSI values are in the same range as earlier reported in multiple studies for various locations (see Supplementary Fig. 3a–d and the Validation section).

### A simple proxy for non-linearity

**Fig. 2:**
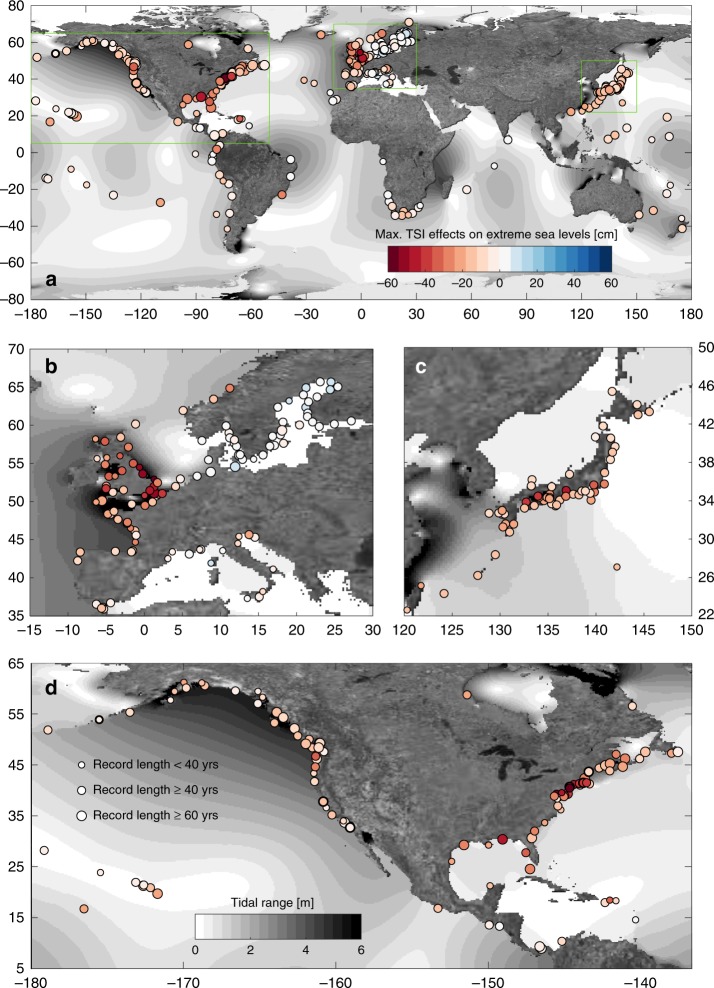
Characteristics of tide and the dependence τ. The subpanels highlight dependencies between (**a**) tide at extreme sea level and non-linear effects, (**b**) dependence τ and non-linear effects, and (**c**) dependence τ and tide at extreme sea level. Non-linear effects <0 causes extreme sea level decreases and non-linear effects >0 increases. Sites dominated by the tide are defined as having a tidal contribution >50%; at sites dominated by non-tidal residual, the non-tidal contribution is >50%.

At 75% of all stations, the tidal contribution to extreme sea levels dominates (i.e., >50%) over the NTR (see Supplementary Supplementary Fig. 4), thus being the main contributor in our analysis (see also earlier work by ref. ^25^). Comparing the proportion of tide [%] vs. the TSI influence [cm] (see Fig. 2a) highlights that average water level reduction (i.e., a negative TSI) is strongest when 60–70% of the extreme sea levels is tidal (e.g., Sandy Hook, USA). TSI approaches zero (or turns positive, i.e., water levels larger than from a linear superposition of tide and NTR alone) if the tidal contribution falls below 20% (e.g., in the Baltic Sea). However, a dependence analysis (see Fig. 2a–c and Supplementary Supplementary Fig. 5) reveals that the correlation between tidal contribution and TSI amounts to only ~0.15. A simple estimate of TSI based on tidal contribution to extreme sea levels alone is thus not suited to robustly estimate the magnitude of TSI. This is emphasised by four selected stations (Rockport, USA; Dutch Harbour, USA; Toulon, France; Puerto de la Luz, Spain) all with TSI ≈ 0 [cm] but tidal contributions ranging from 21–96%, whereas τ only ranges from −0.31 to −0.35. Interestingly, the largest τ values are also found for tidal contributions of 60–70% on average, indicating that large TSI effects are often found in regions where the tidal contribution accounts for nearly two thirds of the total water level but accompanied by a large spread. As a more robust proxy suitable for estimating the influence of TSI effects, we identify τ (at a correlation of −0.83 with TSI induced water level changes) (see Supplementary Fig. 5), a feature which is also visible from the functional dependence highlighted in Fig. 2b.

### Observed temporal changes

**Fig. 3:**
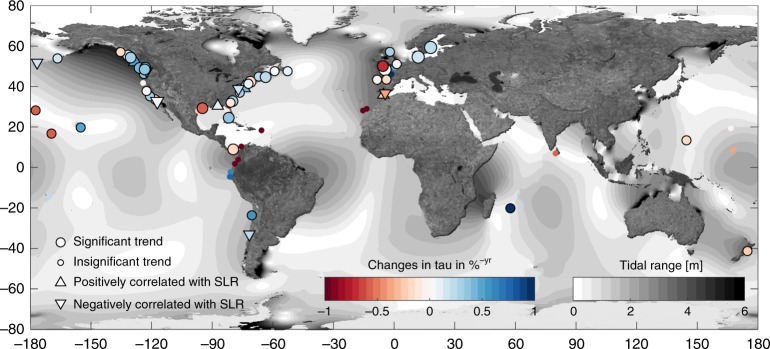
Global map of temporal changes in the dependence τ. Shown are site specific changes in dependence measure τ through time (at least 60 years) with increasing (blue) and decreasing (red) dependencies. Significant trends are shown as bold circles. If the changes are correlated significantly with the observed sea level rise as provided by the permanent service for mean sea level (PSMSL) database, sites are highlighted as triangles.

Changes in τ over the last 60 years or more, as observed at the longest stations on record, are highlighted in Fig. 3. At most stations, including large parts of the hotspots identified above (see Spatial characteristics of non-linear effects section), τ has increased at a significant rate of 2 to 4‰ per year. In consequence, the beneficial effects of TSI (i.e., damping of extreme sea levels) have also grown (see A simply proxy for non-linearity section) since 1950. This leads to a reduction in return water levels, and in turn, in the required coastal defence heights. This relationship could partly remove the need for an enhanced coastal protection ‘allowance’ for the expected increase of extreme sea levels associated with SLR (e.g., ref. ^26^); but a distinct process-based explanation is still missing. Specifically, to estimate the contribution of TSI to future water levels (including extreme sea levels) we first need to understand the driving mechanisms of TSI changes observed in the past. A dependence analysis between the observed τ and SLR resulted in mostly insignificant correlations, and the few significantly correlated stations show both negative and positive dependencies. Moreover, at many stations, significant positive trends in τ appear to be accompanied by significant negative trends in the tidal contribution to extreme sea levels and vice versa (see Supplementary Fig. 6); but again, no clear relationship can be detected. To reliably estimate the contribution of TSI on future water levels, more detailed process-based research on TSI, as well as on tidal characteristics including changes therein are needed (for a comprehensive overview of changes in tides, see ref. ^27^).

### Non-linearity vs. sea level rise

**Fig. 4:**
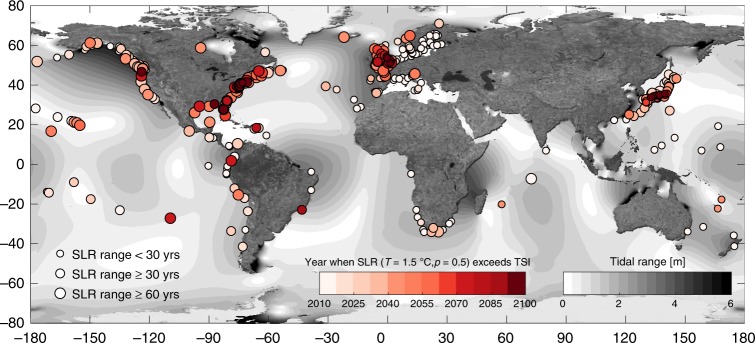
Global map of sea level rise vs. non-linear effects. For all sites, the years when sea level rise (assuming a warming of +1.5 °C by 2100 in global temperatures at the median probability) exceeds non-linear effects (based on the 99th percentile) are shown. The marker size indicates the range (max.-min.) of possible outcomes if other than the median probability sea level rise (i.e., the 5 to 95% probability) projections are used.

At 90% of all stations, we find extreme sea levels (and in turn consequences) to be over-estimated if TSI is not accounted for (see Discussion section). Specifically, Fig. 4 highlights the years at all stations, where TSI effects equal SLR linked to increasing temperatures of +1.5 °C at median probability (50th percentile) (see e.g., ref. ^28^). Again, the aforementioned hotspots of TSI emerge as the most prominent areas showing a TSI effect which corresponds to SLR by up to 2100 at some places. Globally, the average TSI contribution is equal to +1.5 °C (+2.0 °C) SLR projections by 2050 (2040) but this estimate also includes stations where no or even positive TSI effects (i.e., marked by an additional increase) are found. The error introduced by disregarding TSI can thus reach the same order as expected from SLR by 2100, emphasising the need to further develop knowledge of global TSI effects, understand the underlying processes, and integrate TSI in extreme sea level and/or impact assessments.

### Implications of non-linearities for impact assessments

Based on the DIVA modelling framework^1^, the effects of TSI on population and assets exposure are estimated by comparing the 100-year return water levels of ref. ^29^ before and after correcting for TSI effects. TSI is calculated according to Eq. (1), using predictors τ interpolated to all locations of the numerical Global Tide and Surge Reanalysis (GTSR) model of ref. ^29^ as input (see Methods section). At the identified hotspots both the population affected, as well as the costs related to potential flooding by 100-year return water levels decrease noticeably if TSI is considered. In particular, along the entire US coast, TSI causes a reduction of 17% in people affected and reduces direct flood costs of the 100-year event by 13%. Effects of similar order are found for the southern Japanese coast with population and flood costs decreasing by 22 and 15%, respectively; the effects are less pronounced for the UK North Sea coastline at 5 and 7%, respectively. On a global average, the population affected when TSI is considered reduces by roughly 8% and flood costs decrease by almost 16%. This essentially demonstrates two things. First, TSI regulates the physical properties of coastal extremes by reducing the hydrodynamic load on coasts; consequently, the existence of TSI reduces extreme sea levels, compared to the case when the astronomical and non-astronomical components are simply added independently. Second, TSI needs to be considered in global extreme sea level estimates in order to better assess the consequences associated with coastal hazards.

### Validation

A comparison to published TSI values highlights that our results are of similar order. Specifically, ref. ^7^ provided a rough estimate of ~50 cm TSI between Immingham and Cromer on the south-eastern UK coastline. Our statistically derived TSI estimates amount to 55 and 66 cm for these two stations and ~53 cm between them. Further southwest, in the English Channel, our model suggests a maximum TSI of ~42 cm at Dunkerque, ~28 cm at Saint-Malo, and ~18 cm at Le Conquet. Numerical model based investigations focusing on the same region by ref. ^9^ report a TSI of 51–74 cm for Dunkerque, 26–28 cm for Saint-Malo, and 9 cm at Le Conquet. In the Taiwan Strait, a sensitivity study on TSI has been performed with a numerical model by ref. ^8^ with the resulting TSI amounting to ~25 cm at Sansha and ~20 cm at Pingtan station, both located on the northern Fujian coast of mainland China. Our results suggest a TSI of ~27 cm for Sansha and ~25 cm for Pingtan, but due to the lack of freely available tide gauge records at these stations, both values were only indirectly derived using the multiple regression model of Eq. (1) (see Methods) with predictors τ interpolated to all locations of the GTSR model of ref. ^29^. All of these examples are based on individual extreme events, partly also indicating the large spread in the underlying TSI effect; but although being completely independent, they show strong similarities to our results.

Based on a statistical assessment of 220 tide gauge records, ref. ^22^ identified significant TSI in regions previously not investigated such as the Gulf of Panama and the Malayan Peninsula, and smaller but still significant TSI along the North American Pacific Coast and a number of small Pacific Islands (see Supplementary Table 1). Interestingly, all the aforementioned sites are characterised by different bathymetric and geometric features and the authors point to the complicated, site-specific hydrodynamic feedback of TSI. Our assessment confirms the observed spatial patterns of ref. ^22^ but further allows differentiating and quantifying TSI spatially. In particular, along most parts of the Malayan Peninsula, we find an average TSI of 53 cm that increases by up to 61 cm in the north-eastern part and decreases by 48 cm along the western coastline. At the Pacific Islands, we find a rather small TSI of 2–5 cm (see Supplementary Table 1), also consistent with ref. ^22^.

As a cross-validation of our method, we have additionally used the numerical tide-surge model of the German Bight used in refs. ^30,31^ to simulate tide-only, surge-only, and total water levels of 75 extreme events between 1970 and 2013. At the Cuxhaven tide gauge station (i.e., the only German station contained in the GESLA-2 database), TSI has been calculated following the procedure used in ref. ^9^, yielding a numerical model derived TSI of ~30 cm, but ranging from −10 to 85 cm over all events considered. In comparison, the statistically based TSI estimate at Cuxhaven amounts to an average of ~3 cm, a value still reflected in the range of the numerically derived results but also indicating the deficiency of our newly proposed model which is only able to describe average conditions and does not take the observed spread into account. This essentially underpins that TSI effects vary by event and location and have a complicated dependence on wind speed and direction, as well as on the timing of the weather and the tide^7^.

## Discussion

Here we have introduced a novel statistical method to estimate TSI influences on global extreme sea levels based on tide gauge records (see Fig.1). At very shallow locations, where extreme sea levels are a combination of high tides and strong meteorologically-induced surges (such as at Cuxhaven, Germany), our proposed concept to estimate TSI could be misleading as highest observed NTR tends to occur throughout all phases of the tide, thus showing no clear dependence. This is apparent at some stations with TSI ≤ 0 [cm] or τ ≥ −0.35 causing no or even positive TSI effects (see Fig. 2a–c). In our analysis, this applies to ~10% of all sites considered.

Around 75% out of those 10% of all stations with positive TSI effects are found in the Baltic, i.e., a partially enclosed body of water where standing oscillations, commonly referred to as seiches, are often superimposed on extreme sea levels (see e.g., ref. ^32^). Seiche effects are implicitly considered in the marginal distributions used as input to our multivariate statistical analyses, possibly biasing the TSI estimates. However, ref. ^10^ reported a reversed TSI response in a standing oscillation compared to a progressive wave, a feature which has the potential to further increase extreme sea levels. Most of the remaining 25% of all stations with positive TSI effects are located in the tropics and, more interestingly, appear to be also affected by seiches; such as the stations of La Union in El Salvador (ref. ^33^), Fort de France in the Caribbean (ref. ^34^), in the Bay of Paita in Peru (ref. ^35^), or Colombo in Sri Lanka (ref. ^36^). Thus, standing oscillations may at least partly be responsible for the reversed TSI response. Water levels at some places may further be contaminated by local effects (e.g., a damped signal at the location, located in harbours, etc.) or an exceptionally large tidal contribution (see e.g., Fig. 2a–c or the A simple proxy for non-linearity section) both of which are also implicitly included through the marginal distributions.

A statistical method to estimate TSI effects such as presented here is useful to adjust modelled extreme sea levels, derived from superposition of separate tide and surge simulations, towards more realistic conditions. For instance, the GTSR data of ref. ^29^ is a crucial and much needed input for consistent and comparable global risk and impact studies. Comparing differences in the 99th percentile extreme sea levels of GTSR vs. observations shows a mean of ~12.7 cm and a mode of ~10 cm but strongly varying with location and thus potentially biasing the associated risk. After adjusting the GTSR model data for TSI effects using our proposed method, these differences diminish to a mean of ~4.9 cm and a mode of ~6 cm. Excluding stations with τ ≥ −0.35 (i.e., sites contaminated by other than tide and surge only effects such as seiches) further reduces the differences to a mean of ~1.9 cm and a mode of ~4 cm but this also limits the number of usable sites to approximately 90% if applied globally.

For comparison, the differences in 99th percentile extreme sea levels artificially generated by our statistical model vs. observations amount to a mean and mode of ~0 cm, thus being capable of robustly reproducing water level magnitudes. Remaining differences in the adjusted GTSR data can thus be explained by the inability of our TSI regression model to describe conditions that are different from the average (i.e., the underlying spread of TSI vs. τ is not captured in our approach). Further discrepancies may be due to limitations in the applicable range of τ (see above), as well as numerical surge and/or global tide model deficiencies caused by effects other than TSI alone combined in GTSR. However, one needs to keep in mind that the applied GTSR data does not include phenomena other than tide and surge (such as the regional wave set-up). Direct comparability of the adjusted GTSR and observational tide gauge data is thus limited and further research is needed to estimate the extent to which potential other phenomena may explain the remaining differences.

A workaround towards a more realistic representation of extreme sea levels, assuming tide and surge are well represented, is to use a high-resolution numerical model accounting for both tide and surge and their interaction. However, this is still a challenging and computationally expensive exercise, especially at global scale. Our approach provides an alternative way of accounting for TSI and can be combined with independent realisations of tide and surge, contributing to more reliable and consistent risk and impact studies globally.

Our results indicate that NTR and skew surge at highest observed water levels are dependent of the tide and both have not fallen on any extremely high tides. This is a new finding since most existing studies claimed independence between skew surge and the tide but focussed on the tide at the largest skew surges instead. Specifically, the difference between the headline conclusion here and that of ref. ^22^ is that the latter implies an as yet unexperienced storm could occur on any tide (see e.g., Supplementary Fig. 2). Our work suggests that this is maybe not correct—the tide modulates. A future modelling study could, with careful set up, probe these different possibilities further.

Although showing consistent characteristics at all stations considered, our findings can still be biased by the temporally limited input data as severe storm conditions and equinoctial spring tides may simply not yet have been recorded. Consequently, the observed dependency may thus be happenstance or it may be that the highest tides are dynamically influencing the skew surge if using a non-linear approach to their separation.

Here, we highlighted the importance of TSI for coastal impact assessments. Based on a novel statistical method, we identify hotpots where TSI is strongest (i.e., the US East Coast, the UK, and Japan). We show that the effect of TSI at extreme sea levels is in the same order (and also larger at some places) as MSL projections by the end of the century. If TSI is not accounted for in large-scale impact and adaptation studies (as currently done in global studies), and in the decision processes based on their results, the persisting uncertainties from TSI can render important improvements in developing more robust mean sea-level rise projections useless.

## Methods

### Data handling

We use the same dataset as in ref. ^37^ comprising stations of the GESLA-2 database^24^, which are additionally quality checked and spurious outliers or datum shifts etc. are removed before the analysis. All records are interpolated to hourly resolution in order to adjust inconsistencies stemming from different sampling frequencies.

Overall, three sub-sets (medium = M, short = S, long = L) were created. To obtain statistically robust and comparable TSI estimates, we only use records covering at least 30 years and providing water level information to 2010 or later; this results in 362 sites globally (320/42 in the northern/southern hemisphere, respectively) for set M. In a next step, we performed tests on the sensitivity of τ (defined below) against the record length considered. Allowing for a changing τ of up to 1% compared to the longest available observations, we find that records need to cover at least 18 years (comparable to the length of the nodal cycle) on average to provide robust and representative estimates of τ at a site. Consequently, set S contains all records spanning 18 years or more and also ending in 2010 or later. This reduction of the minimum required record length yields a total of 621 sites (480/141), still geographically biased but providing a larger number of sampling points and a more balanced spatial coverage than set M. Finally, set L was constructed to include only those records spanning 60 years or more (i.e., at least two climatological reference periods are considered)^38^, intended to have a long enough span to smooth over climate variations. Again, records in set L were required to end in 2010 or later, reducing the set to a total number of 102 (98/7) stations. Characteristics of all sub-sets, and where they are used throughout this study are summarised in Table 1. Note that the sub-sets will not necessarily contain extreme surge events, and in fact any 18-year record is unlikely to contain the most extreme storms, thereby yielding limited useful information about the occurrence of the most extreme storm surges.

**Table 1:** Data set characteristics and usage.

Used in study	Set	Duration	Stations	End year
A simple TSI proxy	M	≥30 years	362	≥2010
Spatial characteristics of TSI effects	*M_A_	~3300 years	362	–
Validation of TSI estimates	L	≥60 years	102	≥2010
Observed temporal changes	L	≥60 years	102	≥2010
TSI vs. SLR	*M_A_	~3300 years	362	–
Implications of TSI for impact assessment	S	≥18 years	621	≥2010

The table summarises the characteristics of the different sub-sets used in this study, which have been compiled from the GESLA-2 database. All sets have been tailored to address the associated research tasks (see Results section) and differ in duration, the number of stations considered, and the minimum required end year; *indicates artificially generated data (see Methods for more details).

As a general procedure, high-water peaks were extracted and a peak over threshold (POT) method was used to identify extreme events within each set. Previous studies have shown that the POT method best represents the tails of the water level distribution (see e.g., ref. ^39^), thus providing more reliable estimates of extreme sea levels than the block maxima methods (such as the annual maxima (AMAX) method), particularly if records are short^40^. However, selecting an appropriate threshold is still challenging and can bias the statistical assessment at the very beginning. Specifically, if the selected threshold is too low, it causes a bias because the model assumptions are invalid (i.e., values are dependent or non-extreme data are included in the sample). If the threshold is too high, the variance is large because only few data points are included in the analyses^39^. A compilation of different threshold selection criteria is provided in ref. ^41^ concluding that no universally applicable method exists. Here, the largest 3-values/year on average were selected at each individual site ensuring similar sample sizes for all tidal characteristics (e.g., diurnal, semidiurnal). Sensitivity of this choice against our statistical TSI estimates (see Non linearity estimates section) was tested by varying the threshold level between 1-value and 4-value/year on average, showing only minor differences of <1% for 1-value and 2-value, but up to 12% for 4-values/year at the quasi-extreme sea levels.

A declustering scheme of 36 h was applied to ensure independence between individual extreme events, i.e., we sampled each event individually (see e.g., refs. ^42,43^). We are only interested in events driven by a combination of astronomical and meteorological forcing. Therefore, tsunami-induced sea level extremes were removed from the dataset, which could otherwise misrepresent the NTR component derived from a tidal analysis. As a simplified approach, historically recorded tsunamis were compiled and extreme sea levels coinciding with tsunamis (i.e., up to 24 h later) were removed from the analysis.

### Statistical dependence

Estimating the effect of TSI on extreme sea levels is based on separating observational time series in sets M, S, and L into tide and NTR components using the Matlab T_Tide package^44^. The tidal analysis was undertaken for each calendar year using the standard 67 tidal constituents. Tide and NTR at the observed peak high-water levels are extracted and their dependence for all sub-sets is assessed using Kendall’s rank correlation τ which is also required to set up the statistical models (see e.g., ref. ^45^) and in the subsequent regression analysis, described below. Kendall’s rank correlation τ returns values between −1 and 1, where 0 indicates no- and 1 (−1) a perfect relationship (disagreement). For tide and NTR, τ typically ranges between 0 and −1, signifying that as the rank of one variable increases, the rank of the other variable decreases. Lower rank correlations τ (near −1) thus point to a strong relationship, which is equivalent to a pronounced TSI.

Temporal changes in τ are calculated as moving averages during 30-year windows using the longest records (i.e., set L). For each individual site, observed mean sea level (MSL) time series are downloaded from the Permanent Service for Mean Sea Level (PSMSL) website^46^ (https://www.psmsl.org) and compared to changes in τ. Correlation coefficients between MSL and τ are calculated and significance is assessed at the 90% level accounting for serial correlation. Trends are fitted to the time series of MSL and τ using linear regression for the total record lengths available at each site. Confidence intervals are quoted at the 95% confidence level and estimated using a Lag-1 autocorrelation function^41^. Throughout the analysis, we define trends as being statistically significant if they are different from zero at the 95% confidence level.

### TSI estimates

Based on copula functions (i.e., multivariate probability distributions enabling to describe the dependence between random variables), statistically consistent random pairs of tide and NTR are created extending the limited number of observations in set M to a larger set of 10,000 artificial events (corresponding to approximately 3300 years of data at each site), hereafter referred to as set M_A_. Our analyses are based on three different types of copula models including the Frank-, Gaussian-, and t-Copula. The best suited copula model is evaluated by calculating the RMSE between the parametric and the empirical copula, and random events are generated from the parametric copula (including the rank correlation τ) and both marginal distributions. Marginal distributions of both tidal and non-tidal residuals are described by a non-parametric kernel distribution enabling us to fully capture the observed characteristics without introducing a distributional bias. The best copula model for each site is selected by comparing Kendall’s rank correlation τ of the input (i.e., observed tide and NTR) and the simulated data (see Supplementary Fig. 7). For all sites, the model is able to provide artificial events with rank correlation τ differing less than 0.05 (RMSE = 0.017; *r*² = 0.98) and is assumed to adequately represent the observed characteristics.

To assess TSI effects on extreme sea levels, two different copula setups are used. In the descriptive setup (s_d_), observed rank correlations (i.e., τ_d_) of tide and NTR obtained at each location are used as input while the prognostic setup (s_p_) is conducted assuming a zero correlation (i.e., τ_p_ = 0), i.e., no dependence between tide and NTR. Water level percentiles (10th to 90th with increments of ten, as well as the 99th percentile assumed as the maximum water level) of set M_A_ are calculated for setup s_d_ and s_p_ and differences between these are assumed to describe TSI (i.e., TSI = s_p _− s_d_) and its lowering effect on extreme sea levels.

### Regression analysis

We identify an average correlation of R ~0.84 between τ (i.e., tide-NTR dependence) and the TSI induced water level reduction (see Supplementary Fig. 5), a functional dependence, which is described by a multiple regression model as follows:

1 $${\mathrm{TSI}}\sim a + b \, \ast \, \tau + c \, \ast \, \tau ^2 + d \, \ast \, \tau ^3$$

with the observed tide-NTR dependence τ as input and coefficients *a-d* depending on the percentile level (10th–99th) under investigation (see Supplementary Supplementary Table 2, Supplementary Table 3 and Supplementary Fig. 8).

**Fig. 5:**
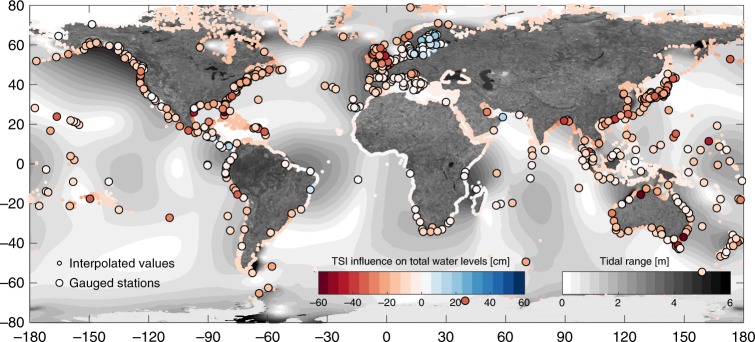
Global map of interpolated non-linear effects (TSIe) influences on extreme sea levels. The figure indicates how changes in non-linear effects (TSIe) extreme sea levels at interpolated (small dots) and observational (bold dots) stations which have been derived using the regression model of Eq. (1). Sites with negative (i.e., reduced extreme sea levels shown red) and positive (i.e., increased extreme sea levels shown blue) non-linear effects are separated according to the colour gradient.

The overall aim of this paper is to provide global estimates of TSI influences on extreme sea levels requiring no further input other than the available water level records. τ values of the shortest but geographically less biased data set S are interpolated to the locations of the water levels used in ref. ^29^ using a natural kriging algorithm, providing estimates of tide-NTR dependence τ for more than 12,000 coastal locations globally (see Supplementary Fig. 9). For all locations, percentile based TSI influences on extreme sea levels are estimated by applying Eq. (1); this is referred to as TSIe (Fig. 5).

### Impact assessment

Estimates of potential impacts due to the inclusion of the TSI effect are based on the Dynamic and Interactive Vulnerability Assessment (DIVA) modelling framework^1^. We use the following two indicators to quantify coastal exposure:

1. Flood costs: damages to assets in the 1-in-100 year floodplain; and
2. Number of people living in the 1-in-100 year floodplain.

We ran the model for present day conditions (2020), first using the 100-year return water levels of ref. ^28^ and second by correcting the data for TSI effects using Eq. (1). Results from the two sets of runs were then compared to quantify the uncertainty introduced by not accounting for TSI in global coastal impact assessments.

### SLR Projections

In 2016, the Paris Agreement of the United Nations entered into force that aims at keeping global temperature rise by 2100 “to well below 2 °C above pre-industrial levels and pursuing efforts to limit the temperature increase to 1.5 °C above pre-industrial levels”^47^, assuming that present-day warming already corresponds to +1 °C compared to pre-industrial conditions^48^. We compare magnitudes of TSI and SLR enabling us to estimate the year when SLR becomes larger than TSI, i.e., the later the year, the larger the uncertainties associated with risk analyses accounting for SLR but neglecting TSI. As input, we use the probabilistic SLR projections of ref. ^28^, which are in line with the Paris Agreement. Their projections include corrections for glacial isostatic adjustment (GIA) (ICE-6G of ref. ^49^), although GIA uncertainty is not considered. All SLR projections are relative to 1986–2005 and available for the median including the 90% uncertainty ranges (5–95%) at 10-year time slices from 2010 to 2100. A resampling to annual SLR values was conducted by using a cubic spline interpolation.

## Supplementary information

Supplementary Information

## Data Availability

The datasets generated during and/or analysed during the study are available from the GESLA website [https://www.gesla.org/].

## Code availability

Codes to generate results that are reported in the paper and central to its main claims are available upon request to the authors.
