# Supplementary material for: Non-linear interaction modulates global extreme sea levels, coastal flood exposure, and impacts
Source: Nat Commun. 2020 Apr 21;11:1918. doi: 10.1038/s41467-020-15752-5 (PMC7174334; doi:10.1038/s41467-020-15752-5)
Supplement: Supplementary file 1 — Supplementary Information [file 41467_2020_15752_MOESM1_ESM.pdf]

Supplementary Information for:

NON-LINEAR INTERACTION MODULATES GLOBAL EXTREME SEA LEVELS, COASTAL FLOOD EXPOSURE, AND IMPACTS

by Arns et al. (2020)

SUPPLEMENTARY FIGURES

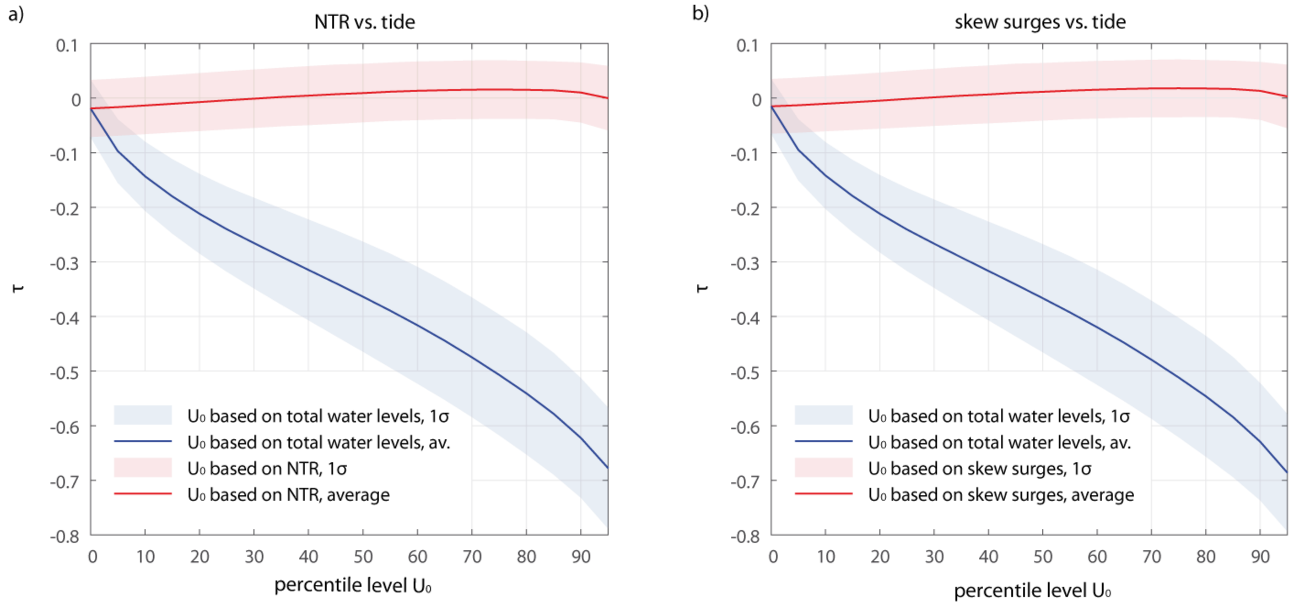

Supplementary Figure 1. Dependence τ vs. percentile level. The figure highlights that both tide vs. non-tidal residual a) and tide vs. skew surge b) dependences τ are strongly related to the total water level percentile level U_0 under investigation. At higher total water level percentiles, the dependence for both non-tidal residual and skew surge vs. tide increases and no marked differences between a) and b) can be detected.

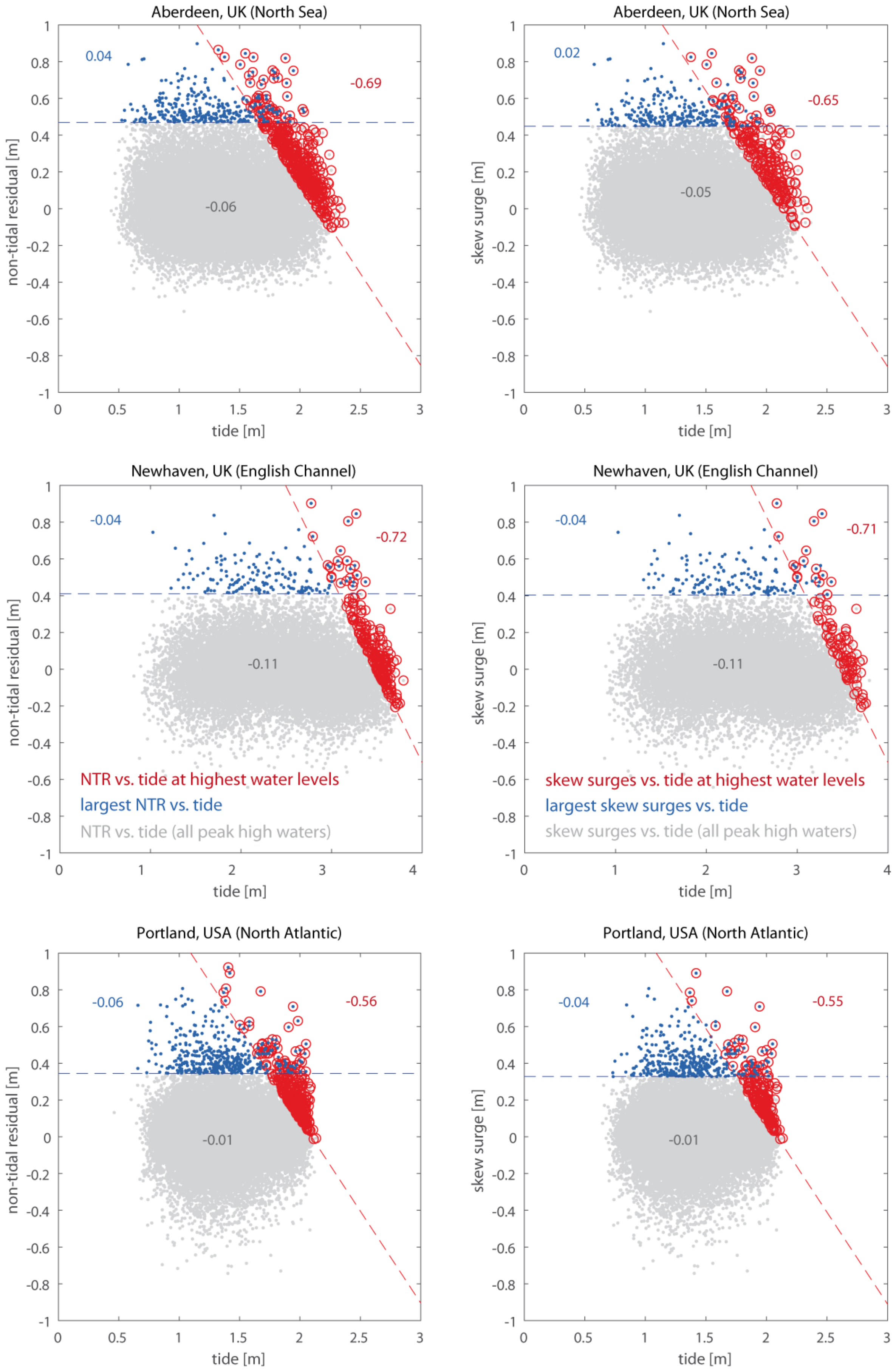
Aberdeen, UK (North Sea)
Newhaven, UK (English Channel)
Portland, USA (North Atlantic)
non-tidal residual [m]
skew surge [m]
tide [m]
0.04
-0.69
-0.06
0.02
-0.65
-0.05
-0.04
-0.72
-0.11
-0.04
-0.71
-0.11
-0.06
-0.56
-0.01
-0.04
-0.55
-0.01
NTR vs. tide at highest water levels
largest NTR vs. tide
NTR vs. tide (all peak high waters)
skew surges vs. tide at highest water levels
largest skew surges vs. tide
skew surges vs. tide (all peak high waters)

Supplementary Figure 2. Site and case dependent correlation. The coloured numbers in the subpanels highlight the dependence τ of non-tidal residual (left) or skew surges (right) vs. tide at the highest water levels (red), largest non-tidal residual (left) or largest skew surges (right) vs. tide (blue), and non-tidal residual (left) or skew surge (right) vs. tide (all total high water peaks) (grey) for three different stations. Although showing minor differences, the general patterns using non-tidal residual or skew surge are similar and no differences in the posterior statistical model (see TSI ESTIMATES) are expected.

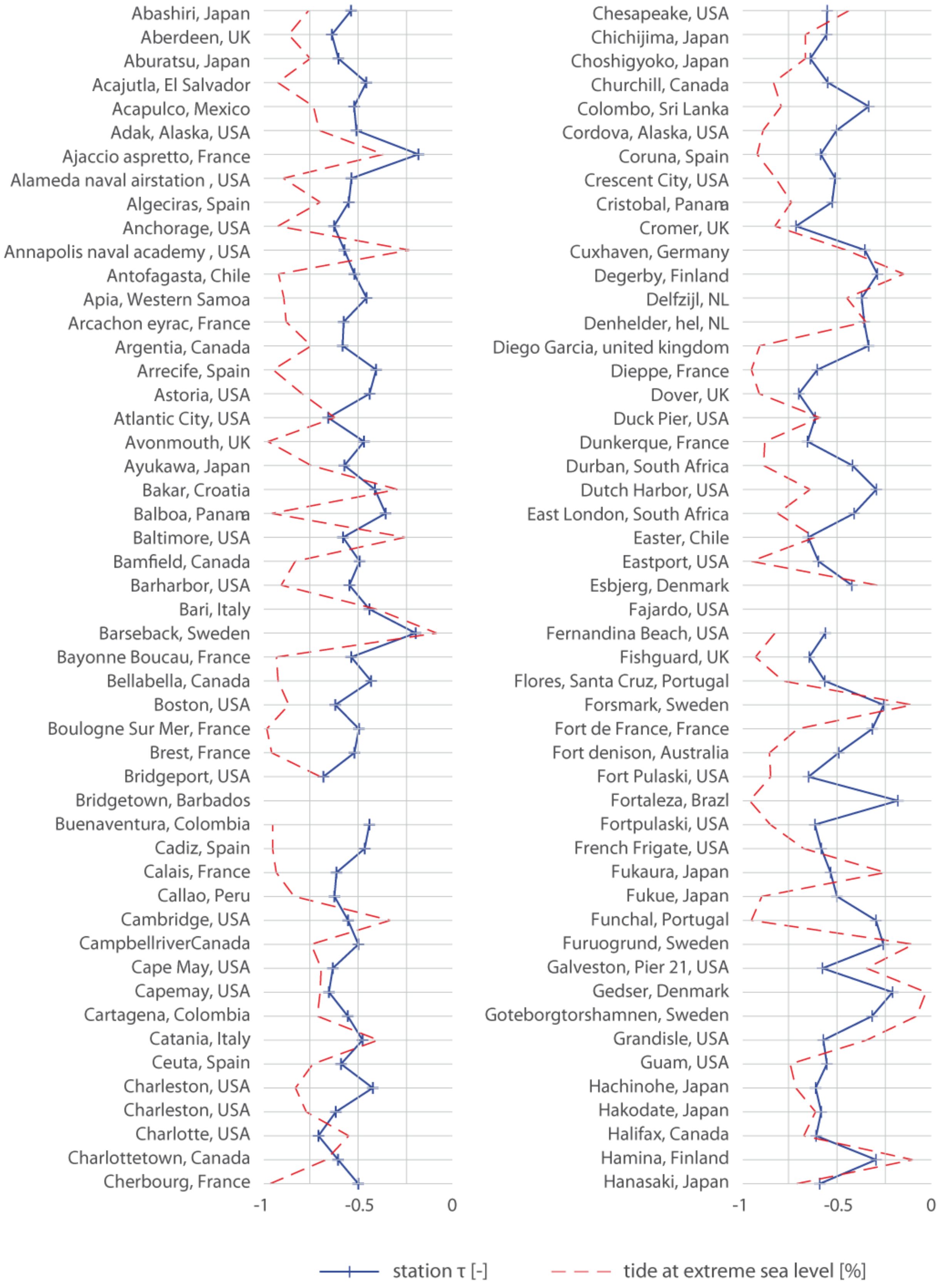

Supplementary Figure 3a. Station based dependence τ and tide at extreme sea level. Shown are site-specific τ values (blue) and the tidal contribution to total observed extreme sea levels (red) as calculated from the observational data.

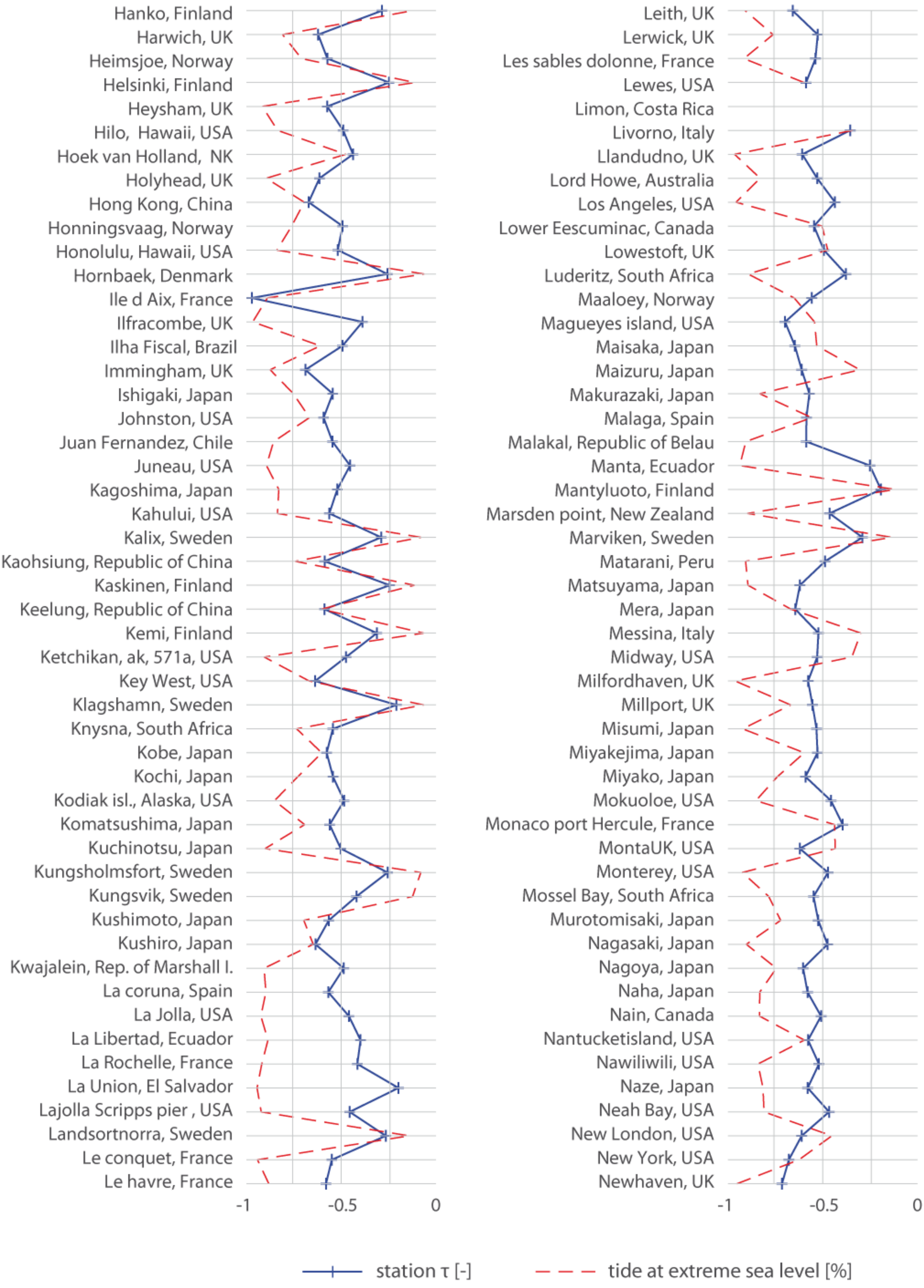

Supplementary Figure 3b. Station based dependence τ and tide at extreme sea level. Shown are site-specific τ values (blue) and the tidal contribution to total observed extreme sea levels (red) as calculated from the observational data.

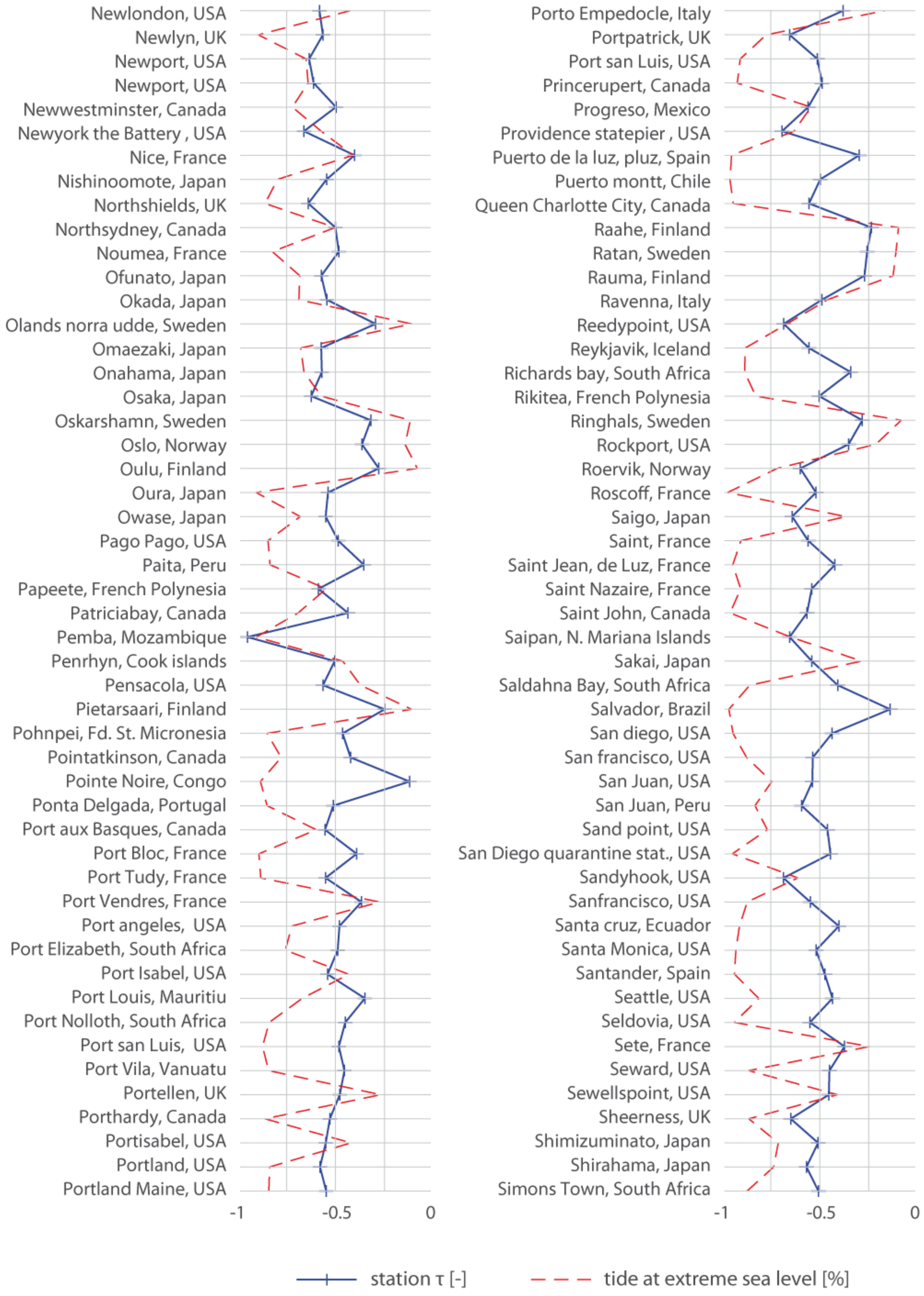

Supplementary Figure 3c. Station based dependence τ and tide at extreme sea level. Shown are site-specific τ values (blue) and the tidal contribution to total observed extreme sea levels (red) as calculated from the observational data.

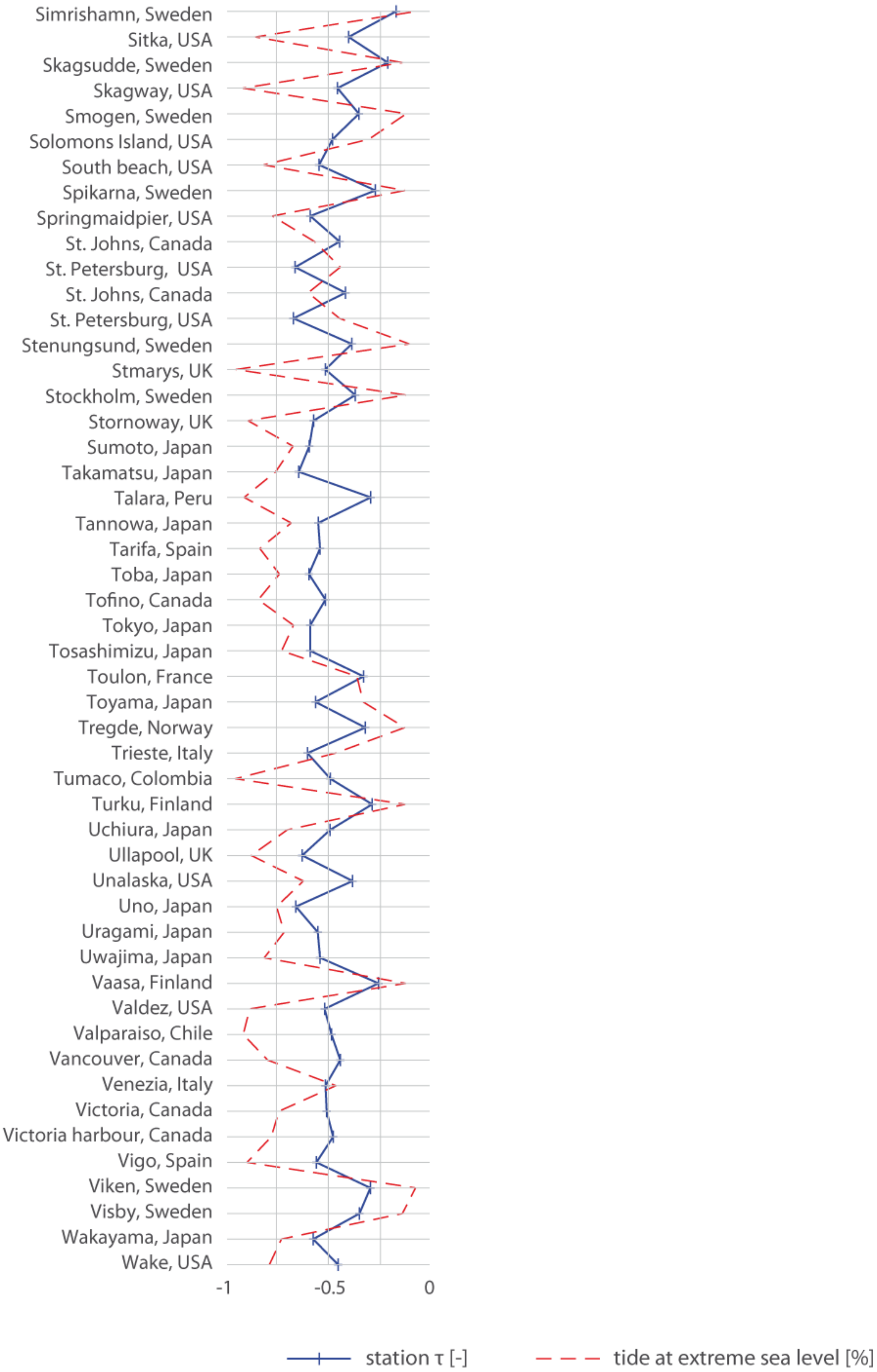

Supplementary Figure 3d. Station based dependence τ and tide at extreme sea level. Shown are site-specific τ values (blue) and the tidal contribution to total observed extreme sea levels (red) as calculated from the observational data.

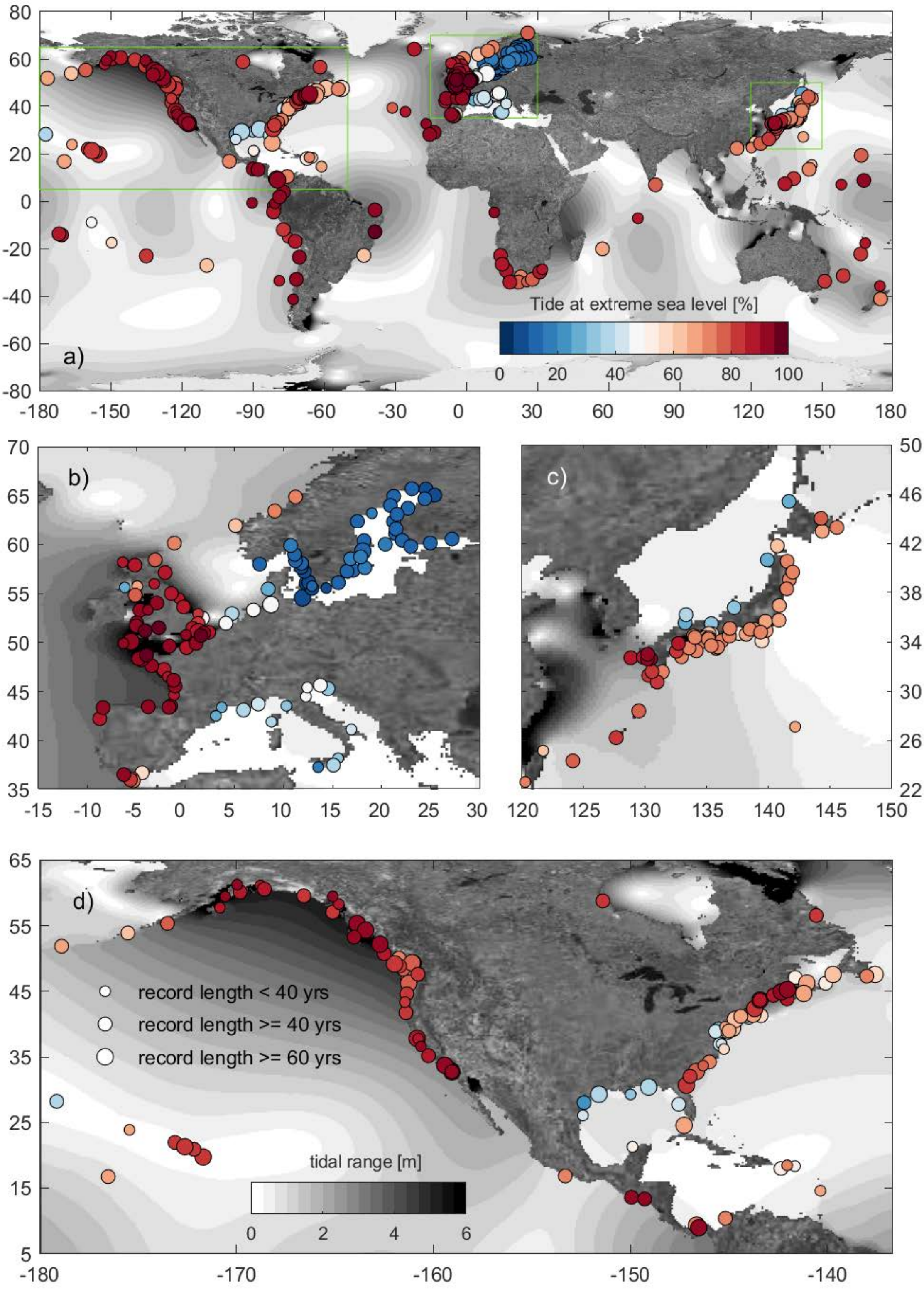

Supplementary Figure 4. Global map of tide at extreme sea levels. Shown are site-specific contributions of tides to extreme sea levels (defined here as the 99th percentile threshold exceedances) for a) the entire World, b) Europe, c) Japan, and d) the USA; Stations can roughly be separated into non-tidal dominated sites (blue) and tide dominated sites (red).

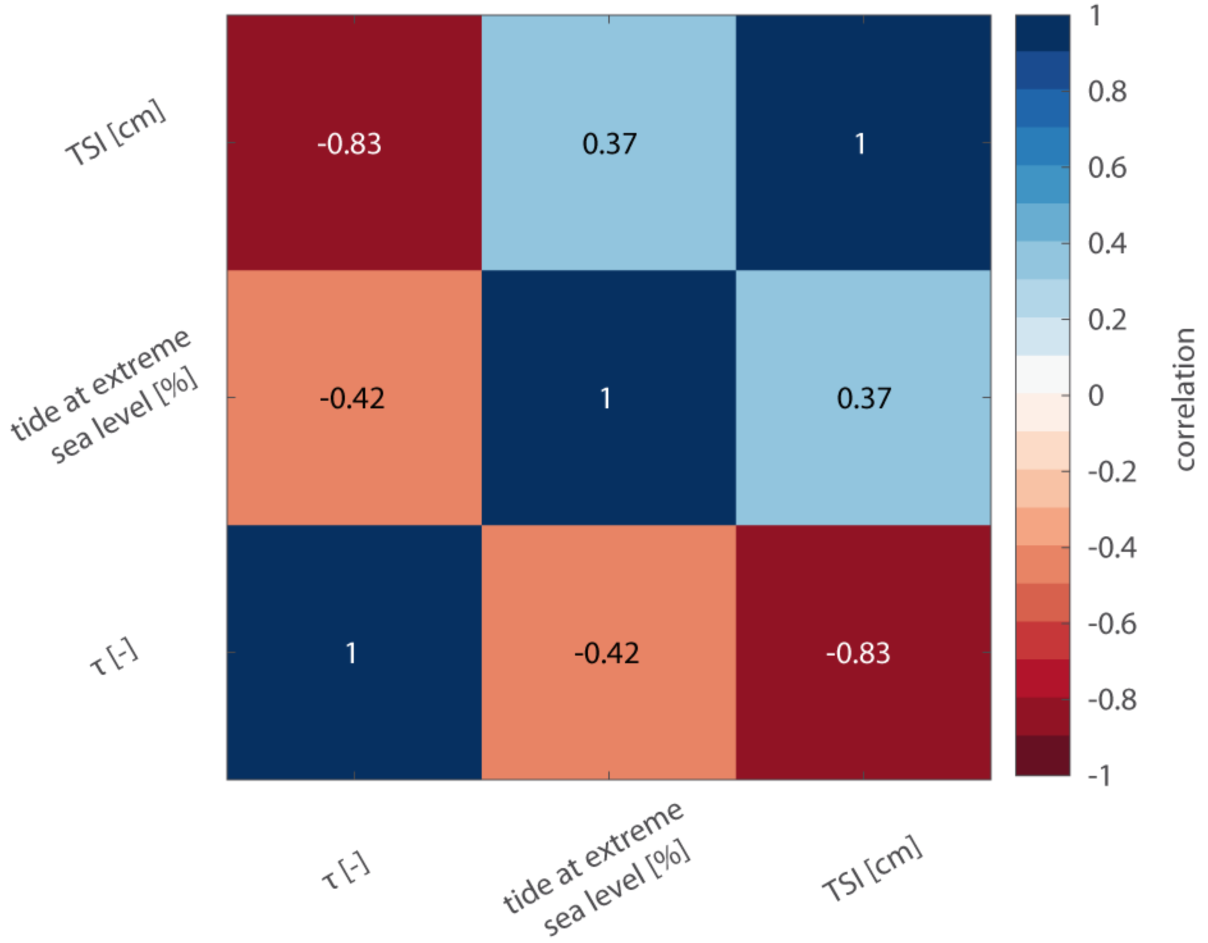

Supplementary Figure 5. Correlation map. Correlation of tide vs. non-tidal residual dependence τ, tide at extreme sea level and non-linear effects (TSI).

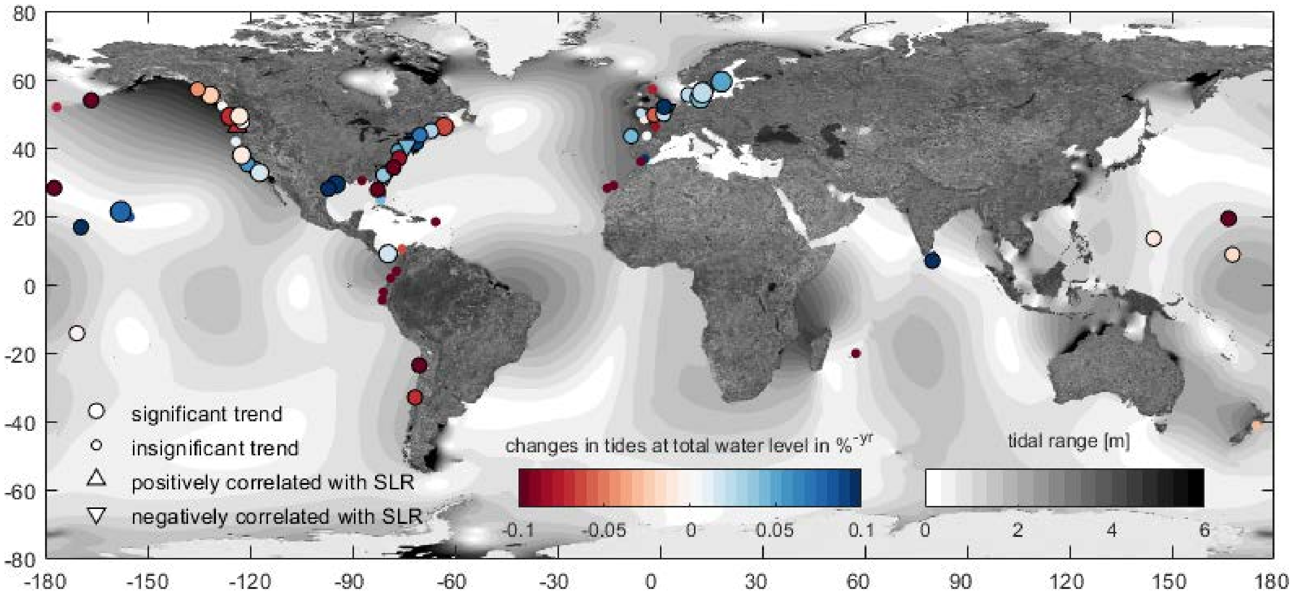

Supplementary Figure 6. Global map of temporal changes in tides. Shown are site specific changes in the relative contributions of tides to extreme sea levels through time (at least 60 years) with increasing (blue) and decreasing (red) tidal contributions. Significant trends are shown as bold circles. If the changes are significantly correlated with the observed sea level rise as provided by the permanent service for mean sea level (PSMSL) database, sites are highlighted as triangle.

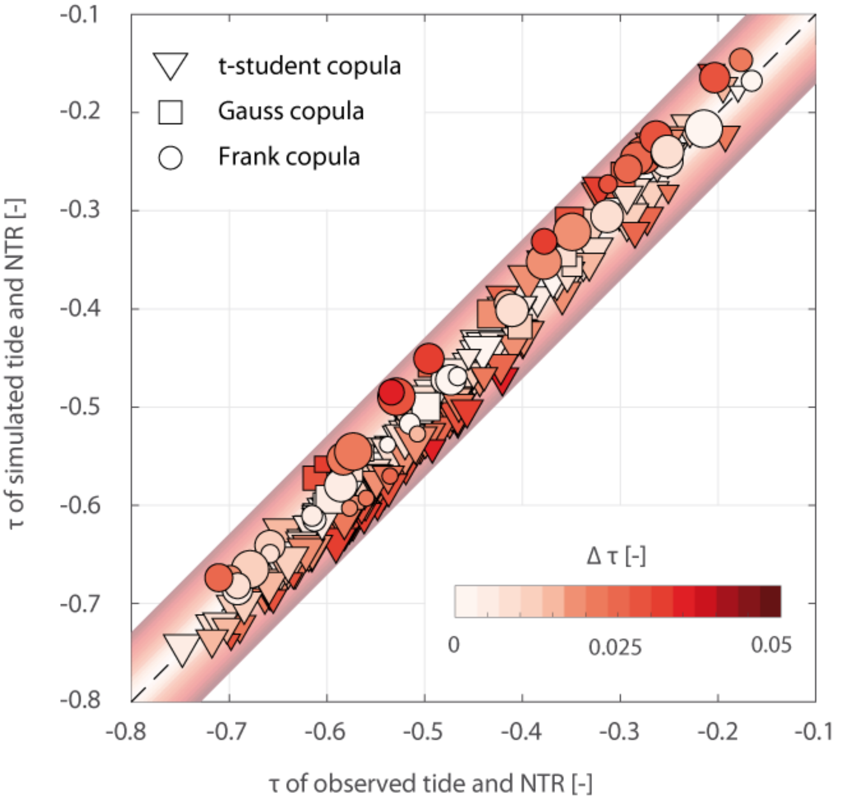

Supplementary Figure 7. Observed vs. simulated dependence τ. Shown are simulated vs. observed dependencies τ between tide and non-tidal residual for the different copula models considered (highlighted by different markers as given in the legend). Increasing differences in τ are emphasized with stronger colours.

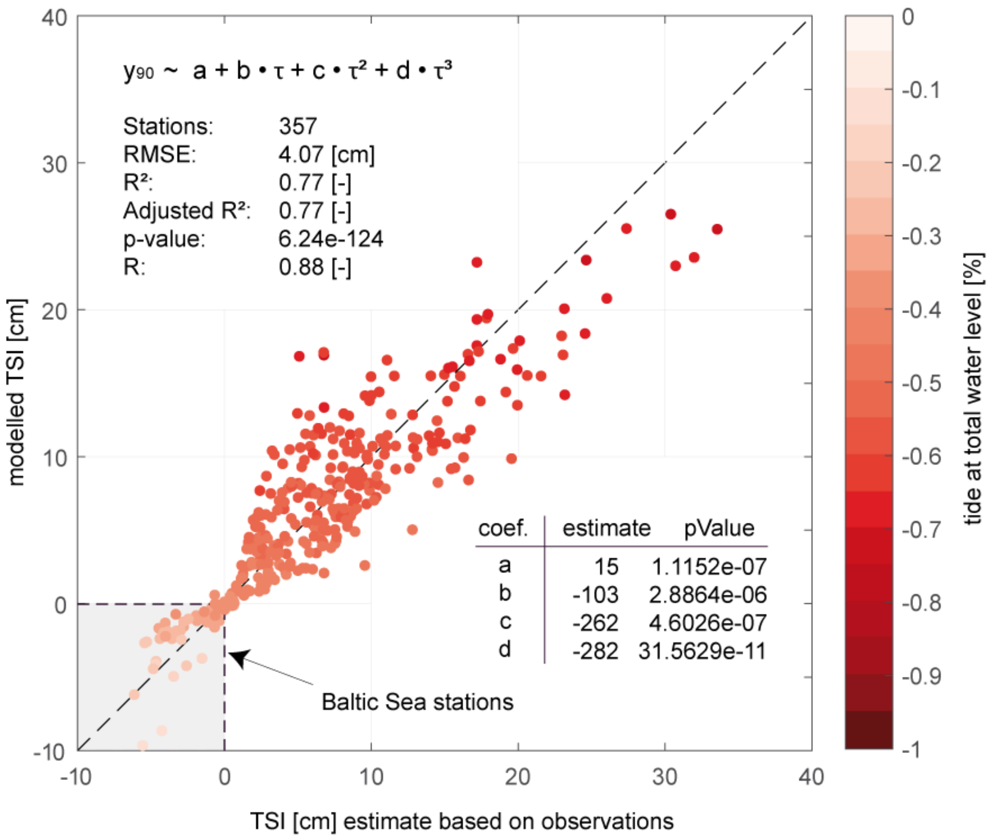

Supplementary Figure 8. Model performance. The performance of observed vs. artificially generated non-liner effects using the proposed regression model is exemplarily highlighted for the 99th percentiles of extreme sea levels. Note that absolute numbers of non-linear effects are given on the y-axis.

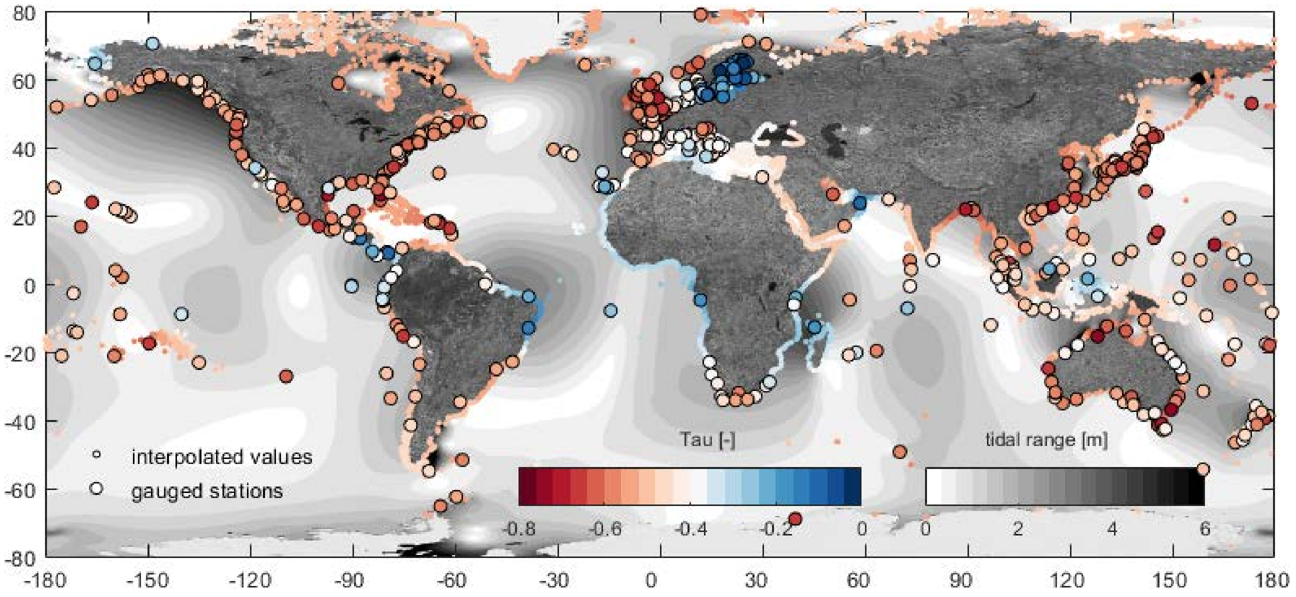

Supplementary Figure 9. Global map of interpolated dependence τ. Shown are interpolated τ values (small dots) which have been derived using the observed dependencies τ (bold dots) as initial situation. Sites with low (blue) and high (red) dependencies are separated according to the colour gradient.

Tab.S1. TSI at small Pacific Islands. The table shows the non-linear effects (TSI) at a number of tide gauges, which are located in the Pacific Ocean. Stations marked with '*' were also used in ref. 22.

Station	Region	TSI [cm]	τ	Tide at extreme sea level [%]
Yap, Fd. St. Micronesia	Western Pacific	5	-0.60	0.83
Midway, USA	Northwestern Pacific	4	-0.53	0.35
French Frigate, USA	Northern Pacific	4	-0.58	0.68
Wake, USA	Western Pacific	4	-0.45	0.79
Guam, USA	Western Pacific	4	-0.55	0.75
Papeete, French Polynesia*	Southern Pacific	3	-0.61	0.57
Penrhyn, Cook islands	Southern Pacific	3	-0.52	0.48
Pohnpei, Fd. St. Micronesia*	Western Pacific	3	-0.48	0.89
Pago Pago, USA	Southern Pacific	3	-0.51	0.89
Port Vila, Vanuatu	Southern Pacific	3	-0.47	0.88
Kwajalein, Rep. of Marshall I.	Southern Pacific	2	-0.50	0.93
Rikitea, French Polynesia	Southern Pacific	2	-0.50	0.84
Apia, Western Samoa	Western Pacific	2	-0.46	0.89

Tab.S2. Coefficients of the multiple regression model. The table shows the coefficients of the regression model explained by eq. (1). Parameters a-d are based on different percentile levels, all of which are referred to the 3 values/yr. on average threshold which has been applied to all datasets of this study. Estimates of TSI in [cm] can be derived by inputting the parameters of Tab.S2 and the tide-NTR dependence τ into eq. (1). SE shows the standard errors of the estimated coefficients. In column t, t-statistics are highlighted and used for making inferences about the regression coefficients based on the hypothesis test on coefficient expressed by parameter p, with values near zero assuming that the corresponding term is significant.

Perc.	a				b				c				d			
	Coef.	SE	t	p	Coef.	SE	t	p	Coef.	SE	t	p	Coef.	SE	t	p
10	-1	0.3	-4.7	0	-10	2.3	-4.3	0	-24	5.5	-4.3	0	-23	4.2	-5.4	0
20	-3	0.6	-4.3	0	-18	4.8	-3.7	0	-41	11.5	-3.5	0	-40	8.7	-4.6	0
30	-4	0.9	-4.6	0	-28	7.3	-3.9	0	-63	17.6	-3.6	0	-62	13.3	-4.6	0
40	-6	1.3	-5.0	0	-40	9.8	-4.1	0	-90	23.6	-3.8	0	-87	17.8	-4.9	0
50	-8	1.6	-5.0	0	-52	12.5	-4.2	0	-119	30.1	-4.0	0	-116	22.8	-5.1	0
60	-10	2.0	-5.0	0	-63	15.4	-4.1	0	-147	37.2	-3.9	0	-145	28.1	-5.1	0
70	-12	2.4	-4.8	0	-75	18.6	-4.0	0	-178	44.8	-4.0	0	-178	33.9	-5.3	0
80	-14	2.9	-4.7	0	-91	22.5	-4.1	0	-222	54.2	-4.1	0	-226	41.0	-5.5	0
90	-15	3.7	-4.1	0	-103	28.9	-3.6	0	-262	69.5	-3.8	0	-282	52.6	-5.4	0
91	-16	3.9	-4.1	0	-106	30.0	-3.5	0	-270	72.2	-3.7	0	-292	54.6	-5.3	0
92	-16	4.0	-4.0	0	-108	31.3	-3.4	0	-277	75.4	-3.7	0	-301	57.1	-5.3	0
93	-16	4.2	-3.9	0	-111	32.7	-3.4	0	-287	78.6	-3.6	0	-314	59.5	-5.3	0
94	-17	4.4	-3.8	0	-115	34.4	-3.3	0	-305	82.8	-3.7	0	-335	62.6	-5.4	0
95	-17	4.6	-3.7	0	-120	36.0	-3.3	0	-323	86.7	-3.7	0	-356	65.6	-5.4	0
96	-18	4.9	-3.7	0	-131	38.3	-3.4	0	-356	92.1	-3.9	0	-391	69.7	-5.6	0
97	-20	5.5	-3.6	0	-144	42.9	-3.4	0	-393	103.3	-3.8	0	-430	78.1	-5.5	0
98	-21	6.4	-3.3	0	-151	49.5	-3.0	0	-419	119.2	-3.5	0	-463	90.2	-5.1	0
99	-23	7.9	-2.9	0	-164	61.5	-2.7	0	-461	148.0	-3.1	0	-517	112.0	-4.6	0

Tab.S3. Test Statistic of the Multiple Regression Model. The table shows the root mean square error (RMSE), the adjusted squared r (r^2), and the explained variability or correlation (r) of the regression model explained by eq. (1). All values are referred to the percentile levels given in the table.

perc.	RMSE [cm]	r^2	r
10	0.32	0.71	0.84
20	0.68	0.71	0.84
30	1.03	0.72	0.85
40	1.38	0.73	0.86
50	1.76	0.74	0.86
60	2.18	0.74	0.86
70	2.62	0.75	0.87
80	3.17	0.76	0.87
90	4.07	0.77	0.88
91	4.23	0.77	0.88
92	4.42	0.77	0.88
93	4.61	0.77	0.88
94	4.85	0.77	0.88
95	5.08	0.77	0.88
96	5.40	0.77	0.88
97	6.05	0.75	0.87
98	6.98	0.73	0.85
99	8.67	0.69	0.83
